# Pseudoprogression of thoracic tumor after radiotherapy in the era of immunotherapy: a case series

**DOI:** 10.3389/fonc.2023.1021253

**Published:** 2023-07-28

**Authors:** Yongbo Xiang, Wei Tang, Jianyang Wang, Zhijie Wang, Nan Bi

**Affiliations:** ^1^ Department of Radiation Oncology, Cancer Hospital, Chinese Academy of Medical Sciences and Peking Union Medical College, Beijing, China; ^2^ Center for National Cancer, Cancer Hospital, Chinese Academy of Medical Sciences and Peking Union Medical College, Beijing, China; ^3^ Department of Medical Oncology, Cancer Hospital, Chinese Academy of Medical Sciences and Peking Union Medical College, Beijing, China

**Keywords:** pseudoprogression, thoracic tumor, radiotherapy, immunotherapy, tumor markers

## Abstract

Pseudoprogression is rarely mentioned after radiotherapy except for central nervous system tumors. With the widespread of immunotherapy, the incidence of pseudoprogression of thoracic tumor after radiotherapy is increasing. This study summarized the clinical features of pseudoprogression in 4 patients who had underwent thoracic radiotherapy after and/or followed by immunotherapy. All of them had received chemotherapy and immunotherapy before thoracic radiotherapy. After radiotherapy, pseudoprogression occurred within 3 months after initiation of immune consolidation/rechallenge therapy. At least a 20% increase in the sum of the longest diameter of target lesions were measured on their chest image. During this period, patients’ ECOG PS scores remained stable, specific serum tumor markers did not increase significantly. Treatment strategies did not change after pseudoprogression. The causes of radiographic pseudoprogression in this case series may be attributed to disturbances such as pneumonitis, atelectasis, mucus blockages and infection. In the era of immunotherapy, pseudoprogression of thoracic tumors after chest radiotherapy might become a common phenomenon. It is important for us to identify pseudoprogression based on patient’s general status, radiological changes, and laboratory tests.

## Introduction

With the rapid development of molecular biology and tumor immunology, immunotherapy has been widely used in multiple tumors. Immune checkpoint inhibitors (ICIs) targeting the programmed cell death protein 1 (PD-1) and its ligand (PD-L1), were widely used for immunotherapy. However, it has been reported that the application of ICIs may cause a rapid increase of tumor load, and then gradually recover to stable state, which is called “pseudoprogression” (1).

Immunotherapy-related pseudoprogression was initially reported in melanoma (2), approximately 7.4% of patients with malignant melanoma who received Pembrolizumab experienced pseudoprogression (3). With the vigorous development of immunotherapy, pseudoprogression have been reported in more and more tumors, it presented in all cancer types except for head and neck squamous cell carcinoma and tumors with mismatch repair deficiency (4). The incidence of pseudoprogression is 0-6% in metastatic non-small cell lung cancer (NSCLC) during ICIs treatment (5). And pseudoprogression may be associated with survival benefit in NSCLC patients. Previous studies revealed that PFS was significantly longer in patients with pseudoprogression than those without pseudoprogression (6).

In the field of radiotherapy (RT), pseudoprogression is commonly described in CNS tumors like glioma (7). It refers to the fact that during postoperative adjuvant RT or concurrent chemoradiotherapy (cCRT) for glioma, some patients show tumor recurrence or progression on brain imaging, but gradually disappear or remain stable after the treatment (7–10). Pseudoprogression occurs in 9%~30% of gliomas during treatment (11). The imaging and clinical features of pseudoprogression and early true progression are very similar thus difficult to distinguish, but the treatment and prognosis of these two conditions are quite different. Pseudoprogression is relatively common in glioma, but rarely reported after thoracic tumor radiotherapy. Based on the patterns of response under immunotherapy, we proposed the concept of pseudoprogression of thoracic tumor after RT in the era of immunotherapy

In this case series, we presented 4 patients who had underwent thoracic RT and anti PD-1/PD-L1 immunotherapy. All of them had experienced pseudoprogression during treatment.

## Case presentations

We reviewed the medical documents of 4 patients with thoracic tumors between 2018 and 2022 in Cancer Hospital, Chinese Academy of Medical Sciences. There were 2 female and 2 male patients with a mean age of 68.5 years (range 61–76, **Table 1**). 3 of these tumors were primary lung cancer and 1 of them was endometrial metastasis. All 4 patients had received thoracic radiation therapy after immunotherapy or followed by consolidation immunotherapy.

**Table 1:** Patient characteristics.

Case No	Age at diagnosis	Diagnosis	Tumor Staging	Initial treatment	RT/CCRT	ICI after RT	Follow-up	Metastasis
1	67/F	Left lung adenocarcinoma	T2bN2M0 IIIA	Chemo+ Anti-PD-1	CCRT	Anti-PD-L1	24 months	–
2	61/M	Left lung squamous carcinoma	T3N3M0 IIIC	Chemo+ Anti-PD-1	RT	Anti-PD-L1	20 months	–
3	70/M	Left lung adenocarcinoma with liver metastasis	T1cN3M1 IV	Chemo+ Anti-PD-1	RT	Anti-PD-1	48 months	Brain metastasis
4	76F	Endometrial adenocarcinoma with right hilar lymph nodes metastasis	TxNxM1 IV	Chemo+ Anti-PD-1	RT	–	62 months	–

Chemo, chemotherapy; ICI, immune checkpoint inhibitor; RT, radiotherapy; CCRT, concurrent chemoradiotherapy.

All 4 patients received regular treatment and follow-up in one institution. Patient’s documents like diagnosis, chemotherapy, RT, immunotherapy, chest imaging, serum tests and other data were obtained from the hospital information System. The therapeutic response was evaluated based on thoracic computed tomography (CT). CT machines used in our study including GE MEDICAL SYSTEMS Discovery CT750 HD, GE MEDICAL SYSTEMS Optima CT660 and SIMENS SOMATON go.top. The image matrix size is 512×512. The spatial resolution is 0.625mm×0.625mm. The slice thickness is 1.0mm and the slice interval is 0.8mm. All patients underwent helical CT scan. Measurement of primary lung tumor and mediastinal lymph node was on lung window (WW/WL, 1500/-550HU) and mediastinal window (WW/WL, 380/35HU), respectively. We focused on the radiological variation of target lesions before and after RT, during and after pseudoprogression.

Tumor treatment response was evaluated base on Response Evaluation Criteria In Solid Tumors (RECIST 1.1) criteria and Immune Response Evaluation Criteria In Solid Tumors (iRECIST) criteria. According to RECIST 1.1 criteria, progressive disease (PD) was defined when at least a 20% increase in the sum of the longest diameter of target lesions, taking as reference the smallest sum longest diameter recorded, or the appearance of new lesions. No further imagining reexamination is required for PD confirmation in RECIST 1.1. iRECIST are based on RECIST 1.1, immune unconfirmed progressive disease (iUPD) is defined as the initial evaluation of PD based on RECIST 1.1. However, immune confirmed progressive disease (iCPD) requires further imaging reassessment, which is done 4 weeks or later. Progressive will be confirmed only if target lesions enlarge or new lesions appear.

In addition to the thoracic CT, The Eastern Cooperative Group Performance Status (ECOG-PS) scores, serum tumor markers were also recorded throughout the treatment process. Since the positive expression rate of carcinoembryonic antigen (CEA) increased significantly in lung adenocarcinoma, and squamous cell carcinoma antigen (SCC) in lung squamous cell carcinoma and CA-125 in endometrial carcinoma. These 3 serum tumor markers were also applied to assess treatment response.

### Case 1

A 67-year-old woman who had nodules in the upper lobe of her right lung was pathologically diagnosed as pulmonary adenocarcinoma with mediastinal lymph node metastasis (T2N2M0, stage IIIA). The patient received cCRT of 60Gy after 2 cycles of induction chemotherapy plus anti-PD-1 immunotherapy (sintilimab). Chest CT scan revealed partial remission (PR) of target lesions according to RECIST 1.1 criteria. Then she received consolidation Durvalumab 1 month after the end of cCRT. The consolidation treatment had been discontinued due to grade 2 treatment-related pneumonitis, and retreatment with anti-PD-L1 therapy was given after recovering from pneumonitis. 1 month after rechallenge of immunotherapy, her first CT scan reexamination indicated enlargement of the left upper lobe tumor, target lesions were indistinct from the consolidation shadow of the distal lung field lesion. There were no significant changes in mediastinal lymph nodes. Response evaluation revealed PD according to RECIST 1.1, and iuPD according to iRECIST. She remained clinically asymptomatic and tumor marker test from serum like CEA were stable and normal. These evidences suggested the enlargement of target lesions might be pseudoprogression. The patient remained well and continued on anti-PD-L1 treatment during the 24 months of follow up. Subsequent chest image revealed continued shrinkage of the target lesions.

### Case 2

A 61-year-old man with chest tightness was pathologically diagnosed as left pulmonary squamous cell carcinoma (T3N3M0, stage IIIC). Since he couldn’t tolerate cCRT, thoracic RT of 50Gy was performed after 6 cycles of chemotherapy plus anti-PD-1 immunotherapy (Pembrolizumab). Chest CT scan after treatment revealed PR of target lesions. Then he received Durvalumab consolidation 1 month after the end of RT. Chest CT scan 1 month after the immuno-consolidation therapy showed that the proximal lesion of the left lower lobe of the lung was fused with the distal nodules, and the boundary was unclear. This led to the enlargement of target lesion measurement. No significant enlargement of his hilar, mediastinum and supraclavicular lymph nodes has been detected. Thoracic MR at pseudoprogression presented irregular soft tissue enlargement in the left lower lobe, and the enhancement of distal nodule was slightly weaker than that of proximal nodule. The patient had no clinical symptoms and his specific tumor marker like SCC had not increased. Response evaluation revealed PD according to RECIST 1.1, and iuPD according to iRECIST. We considered this phenomenon as pseudoprogression and continued immunotherapy. The patient was in good condition and his tumor did not progress during the 20 months of follow-up.

### Case 3

A 70-year-old man was found to have left lung and liver masses during annual physical examination, he was diagnosed as left pulmonary adenocarcinoma by puncture biopsy, with mediastinum lymph nodes, brain, and liver metastasis (T1cN3M1, stage IV). He received multicourse chemotherapy plus anti-PD-1 antibody (Pembrolizumab) immunotherapy for 12 months, then his primary tumor and 4R lymph-node progressed. The patient received thoracic RT of 60Gy subsequently. Chest CT scan after RT revealed PR of target lesion. He continued Chemotherapy and anti-PD-1 antibody (Pembrolizumab) 3 weeks after the end of RT. 4 months later, chest CT scan showed that the target lesion in the left lower lobe of the lung was enlarged while involved lymph nodes and liver metastases remained stable (iuPD). However, the patient had no significant clinical symptoms and no significant increasement of specific tumor marker like CEA. Therefore, pseudoprogression was considered and he continued to receive immunotherapy. He underwent stereotactic RT 2 months after thoracic RT for brain metastases. His chest tumor remained stable during 48 months of follow-up.

### Case 4

A 75-year-old woman underwent bronchial wedge resection for her right lung nodules. Postoperative pathological analysis revealed lung metastasis from endometrial cancer (TxNxM1, stage IV). She was diagnosed with endometrial carcinoma and underwent surgical resection 5 years ago. 2 years after lung operation, her Chest CT scan reported multiple enlarged lymph nodes in mediastinum and hilum. Metastatic tumor progression was therefore diagnosed. She received lenvatinib combined with anti-PD-1 antibody treatment. Unfortunately, immunotherapy had to be stopped due to significantly elevated lipase after 2 cycles of treatment. Subsequently, she received 6 cycles of second line chemotherapy (paclitaxel + carboplatin), and the tumor was partially remission. The patient received chest RT of 54Gy 1 month after chemotherapy, and the chest lesions were significantly reduced at the end of RT. However, His multiple hilar lymph nodes in CT scan presented to be enlarged 1 month after RT. No other lesions were detected. Response evaluation revealed PD according to RECIST 1.1 criteria, and iuPD based on iRECIST. The patient remained clinically asymptomatic. And there was no significant change of endometrial cancer-associated serum tumor marker like CA125. These evidences suggested that the enlargement of the lymph nodes was pseudoprogressive, and she did not receive any additional treatment. Follow-up CT examination revealed that the enlarged lymph nodes of the chest continued to shrink. Her disease remained control well during 62 months of follow-up.

## Results

As shown in **Table 2**, all 4 patients had received chemotherapy and immune checkpoint inhibitors (ICIs) before thoracic radiotherapy. All patients underwent intensity modulated radiation therapy (IMRT), which is an advanced RT technique used in lung cancer patient. Concurrent chemoradiotherapy was used in case 1. And radiation therapy alone was used in case 2-4. 2 patients had received immune consolidation therapy after thoracic RT while 1 patient had received chemotherapy together with immunotherapy. After RT, 2 patients developed pneumonitis (radiation pneumonitis or immune-associated pneumonitis) and 1 patient developed radiation esophagitis. Pseudoprogression presented in all 4 patients, and the average interval time from consolidation/rechallenge immunotherapy to pseudoprogression was 1.6 months (range from 0.8 to 3 months). Except for one patient who did not use consolidation therapy, pseudoprogression occurred 1 month after the end of RT. All patients encountered at least a 20% increase in the sum of the longest diameter of target lesions based on RECIST 1.1 criteria.

**Table 2:** Timeline of intervention and response evaluation for all patients.

	Patient 1	Patient 2	Patient 3	Patient 4
Initial diagnosis & intervention	- Left lung Adenocarcinoma - Pemetrexed+ DDP - Sintilimab	- Left lung Squamous carcinoma - Abraxane+ DDP; - Pablizumab	- Left lung Adenocarcinoma with liver metastasis -Pemetrexed+ Carboplatin; Docetaxel; Abraxane - Pablizumab	- Endometrial Adenocarcinoma with right pulmonary metastasis - Paclitaxel liposome+ Carpbolatin; - PD-1+Lenvatinib
Changes on chest CT & response evaluation	- Decreasing size of the left upper lobe lesion (3.7×2.8cm) - RECIST 1.1: SD - iRECIST: iSD	- Decreasing size of the left lower lobe lesion (1.9×1.6cm) - RECIST 1.1: PR - iRECIST: iPR	- Increasing size of the left lower lobe lesion (3.3×3.2cm) - RECIST 1.1: SD - iRECIST: iSD	- Multiple enlarged lymph nodes in the right hilum and mediastinum, the largest were 1.5×0.9cm - RECIST 1.1: SD - iRECIST: iSD
Chest RT/CCRT	60Gy	50Gy	60Gy	54Gy
Changes on Chest CT & response evaluation after RT	- Decreasing size of the left upper lobe lesion (3.0×1.8cm) - RECIST 1.1: SD - iRECIST: iSD	- Decreasing size of the left lower lobe lesion (1.4×0.9cm) - RECIST 1.1: PR - iRECIST: iPR	- Decreasing size of the left lower lobe lesion (2.1×1.2cm) - RECIST 1.1: PR - iRECIST: iPR	- Decreasing size of enlarged lymph nodes in the right hilum and mediastinum, the largest were 1.0×0.5cm - RECIST 1.1: PR - iRECIST: iPR
Pseudoprogression	- Incresing size of the left upper lobe lesion (3.9×3.8cm) - RECIST 1.1: PD - iRECIST: iUPD - clinical symptoms were stable	- Fusion of the left lower lobe lesion and distal nodule (4.4×2.5cm) - RECIST 1.1: PD - iRECIST: iUPD - clinical symptoms were stable	- Increasing size of the left lower lobe lesion (5.4×3.0cm) - RECIST 1.1: PD - iRECIST: iUPD - clinical symptoms were stable	- Increasing size of enlarged lymph nodes in the right hilum and mediastinum, the largest were 3.8×1.8cm - RECIST 1.1: PD - iRECIST: iUPD - clinical symptoms were stable
Time from initiation of ICI to pseudoprogreesion	12 months	9 months	6 months	11 months
Time from end of RT to pseudoprogression	9 months	2 months	4 months	1 month
Time from consolidation or rechallenge of immunotherapy to pseudoprogression	1 months	0.8 month	3 months	–
Reassessment after pseudoprogression	- Decreasing size of the left upper lobe lesion (2.8×2.6cm) - RECIST 1.1: PR - iRECIST: iSD	- Fusion of the left lower lobe lesion and distal nodule (4.9×1.8cm) - RECIST 1.1: SD - iRECIST: iSD	- Decreasing size of the left lower lobe lesion (3.9×2.5cm) - RECIST 1.1: SD - iRECIST: iSD	- Decreasing size of enlarged lymph nodes in the right hilum and mediastinum, the largest were 1.2×0.9cm - RECIST 1.1: PR - iRECIST: iSD
Follow-up	- Continued Duvalizumab consolidation therapy - Stable at 24 months after diagnosis	- Continued Duvalizumab consolidation therapy - Stable at 20 months after diagnosis	- Continued Pablizumab consolidation therapy - Brain metastasis was found and treated with RT at 20 months after diagnosis - Still alive at 48 months after diagnosis	- Stable at 62 months after diagnosis

ICI, immune checkpoint inhibitor; RT, radiotherapy; CCRT, concurrent chemoradiotherapy; RT, Radiotherapy; RECIST, Response Evaluation Criteria In Solid Tumors; iRECIST, Immune Response Evaluation Criteria In Solid Tumors; PR, partial remission; SD, stable disease; PD, progressive disease; iUPD, immune unconfirmed progressive disease.

**Figure 1:**
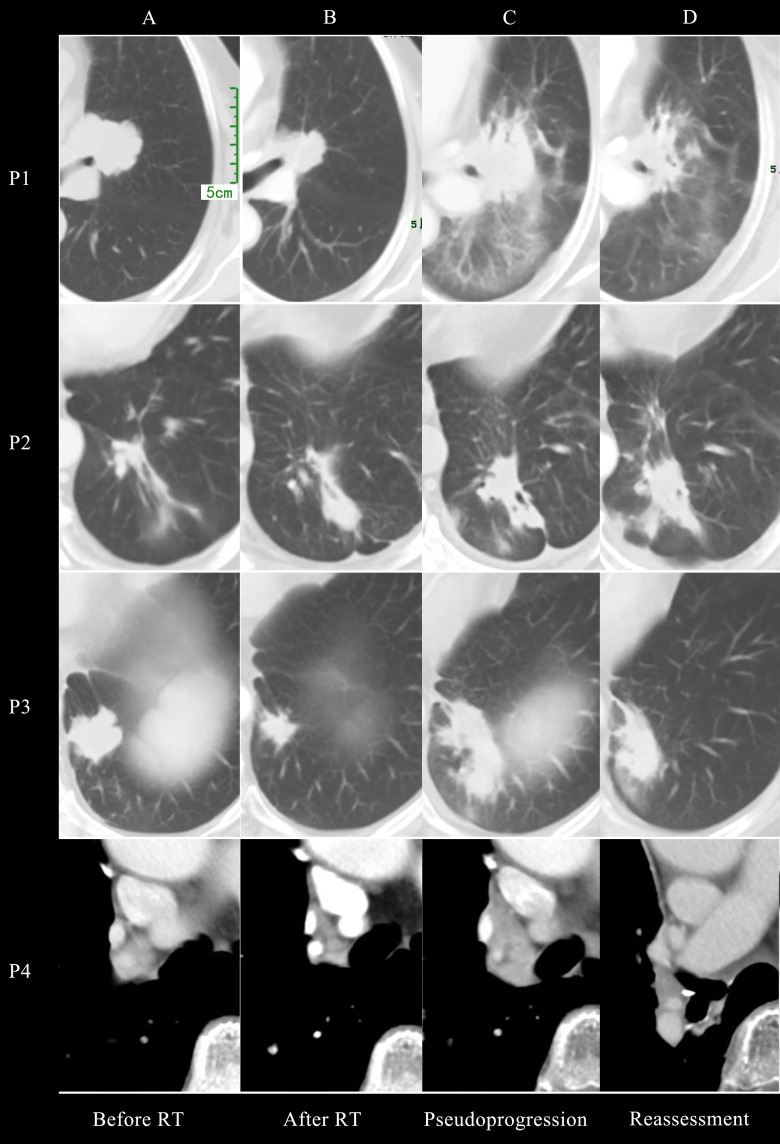
Chest CT imaging of all 4 patients before radiotherapy **(A)**, after radiotherapy **(B)**, at pseudoprogression **(C)** and first reassessment after pseudoprogression **(D)**.

**Figure 1** presented the thoracic radiological features of 4 patients. Before RT, Target lesions were all in PR or stable state. After thoracic RT, the first chest CT assessment showed that the target lesions in all 4 patients were reduced, among which 2 patients were evaluated as PR and 2 as SD. Pseudoprogression occurred at 0.8-3 months after RT or consolidation immunotherapy. According to RECIST 1.1 Criteria, all patients were evaluated as PD, but the target lesions did not significantly increase in further reassessment, therefore the target lesions are rated as iUPD based on iRECIST Criteria. None of the them had encountered an exacerbation of clinical symptoms, nor did they change their treatment regimen due to pseudoprogression.

**Figure 2:**
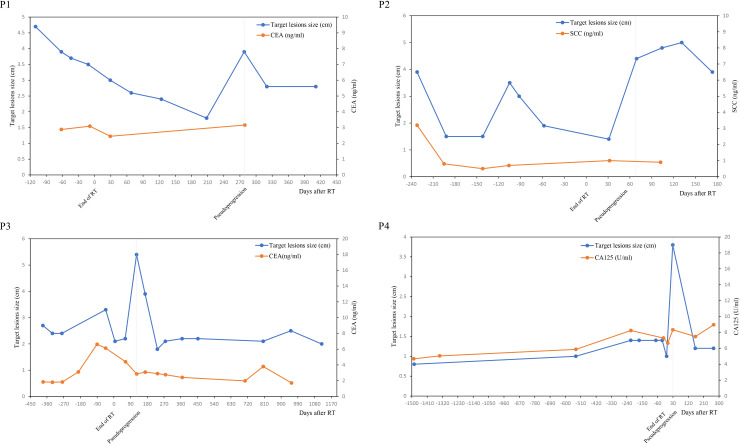
Line chart of target lesions size and serum tumor markers over time.

Target lesions size and serum tumor markers value was shown in **Figure 2**. It was found that the variation trend of serum tumor markers such as CEA, SCC and CA-125 was basically consistent with the variation trend of target lesions size, especially in case N0.2, N0.3 and N0.4. The size of the target lesions increased sharply during pseudoprogression, Nevertheless, Serum levels of tumor markers did not rise concomitantly.

Additionally, all patients’ ECOG PS is 0 or 1 before or after pseudoprogression. And the physical state and general state of them did not deteriorate after RT. Therefore, when tumor progression is first detected on chest CT, the treatment of patients did not change. Subsequent reassessments of chest CT confirmed that the enlargement of the target lesions were pseudoprogression.

## Discussion

Pseudoprogression was initially reported in melanoma, In the field of radiotherapy, pseudoprogression commonly appeared in glioma. The concept of pseudoprogression has not yet been uniformly defined. Here we have reported 4 patients with thoracic tumor who presented with pseudoprogression after RT combined with immunotherapy. All patients had received chemotherapy and ICIs before or after thoracic RT. All of them were assessed as PD or iUPD after RT, and Reexamination ultimately confirmed as pseudoprogression. It may occurred at 0.8-3 months after RT or consolidation/rechallenge immunotherapy. During this period, the patient was generally in good condition, and serum tumor markers did not increase significantly. Therefore, their treatments remained the same.

Pseudoprogression is not true progression of the disease. The mechanism of this phenomenon has not been fully elucidated, and it is mostly considered to be caused by inflammatory cell infiltration, edema, necrosis and delayed effect of immune cells towards tumor during immunotherapy (3, 12–14). In this study, the reasons for pseudoprogression were different among the 4 patients. The first patient developed pneumonia after RT, which may lead to enlarged consolidation shadows around the lesion. The second patient had another nodule distal to the lesion, which may be atelectasis and mucous plug. At first, the two nodules could be measured separately, but after RT, the two nodules gradually fused into a whole, resulting in the enlargement of the lesion measured. The third patient developed both lung pneumonia during pseudoprogression, which may have resulted in increased consolidation around the tumor. The fourth patient presented with transient enlargement of lymph nodes, which may be inflammatory lymph node enlargement caused by infection, but the cause cannot be fully determined with the current data. None of these patients had undergone biopsy, because in clinical practice, rebiopsy is difficult for most patients. If target lesions increase or new lesions are found on imaging, whether to continue the original plan or adjust the treatment plan becomes a difficult problem in clinical treatment. Therefore, it is important to distinguish between pseudoprogression and real progressive disease. A flow chart of differentiating pseudoprogression and real progression has been presented in **Figure S1**.

At present, RECIST or RECIST 1.1 revision are mostly used as the traditional response evaluation criteria for solid tumors (15). However, they are inadequate to distinguish between true progression and pseudoprogression. Therefore, new criteria such as immune related Response Criteria (irRC) (16), irRECIST, iRECIST and imRECIST were introduced (17). The iRECIST standard introduces the concept of iUPD (immune unconfirmed progressive disease) and iCPD (immune confirmed progressive disease), which allows patients with iUPD to continue their current treatment for at least 4 weeks as long as their clinical condition is stable.

There are several emerging approaches for identifying pseudoprogression. In addition to CT and MR, PET can help distinguish between pseudoprogression and true progressions. In small retrospective study involving 5 patients with melanoma with brain metastases, PET showed a distinction between pseudoprogression (low tracer uptake) and true progression (high tracer uptake) (18). In a study of melanoma and NSCLC, 3 patients encountered pseudoprogression during treatment, with imaging findings showing tumor enlargement and a significant decrease in serum IL-8 levels, and serum IL-8 levels remained below baseline when tumor load back to normal. The researchers therefore hypothesized that IL-8 might be a serum marker for the diagnosis of pseudoprogression (19). Another approach uses liquid biopsies to monitor KRAS-mutated chromosomal instability to identify pseudoprogression and quantified circulating tumor DNA (ctDNA) has been shown to decrease in pseudoprogression compared with true progression (4, 20). ctDNA has been shown to be another marker for differentiating pseudoprogression from true tumor progression. However, all the above results are based on small sample size. To date, none of above have been validated to be a convincing method to distinguish pseudoprogression from true progression.

To the best of our knowledge, the concept of pseudoprogression has rarely been mentioned in the field of thoracic RT. We believed that with the continuous broadening of the application of immunotherapy, this phenomenon in thoracic tumor after RT will be more and more common. Since pseudoprogression may be associated with survival benefit, it is important for us to identify pseudoprogression and make further treatment decision accordingly.

## Data availability statement

The raw data supporting the conclusions of this article will be made available by the authors, without undue reservation.

## Ethics statement

Written informed consent was obtained from the individual(s) for the publication of any potentially identifiable images or data included in this article.

## Author contributions

We were all involved in the management of these patient. YX and NB conceptualized the manuscript. YX wrote the original draft and NB wrote the final version. YX, WT and JW provided support in the image measurement. JW, ZW, and NB critically revised the manuscript. Written consent for publication was obtained from all patients. All authors contributed to the article and approved the submitted version.

## Supplementary material

Supplementary Figure 1 A flow chart of differentiating pseudoprogression and real progression. PD, progressive disease; iUPD, immune unconfirmed progressive disease; iCPD, immune confirmed progressive disease.
