# Supplementary figures and images for: Pseudoprogression of thoracic tumor after radiotherapy in the era of immunotherapy: a case series
Source: Front Oncol. 2023 Jul 28;13:1021253. doi: 10.3389/fonc.2023.1021253 (PMC10419187; doi:10.3389/fonc.2023.1021253)

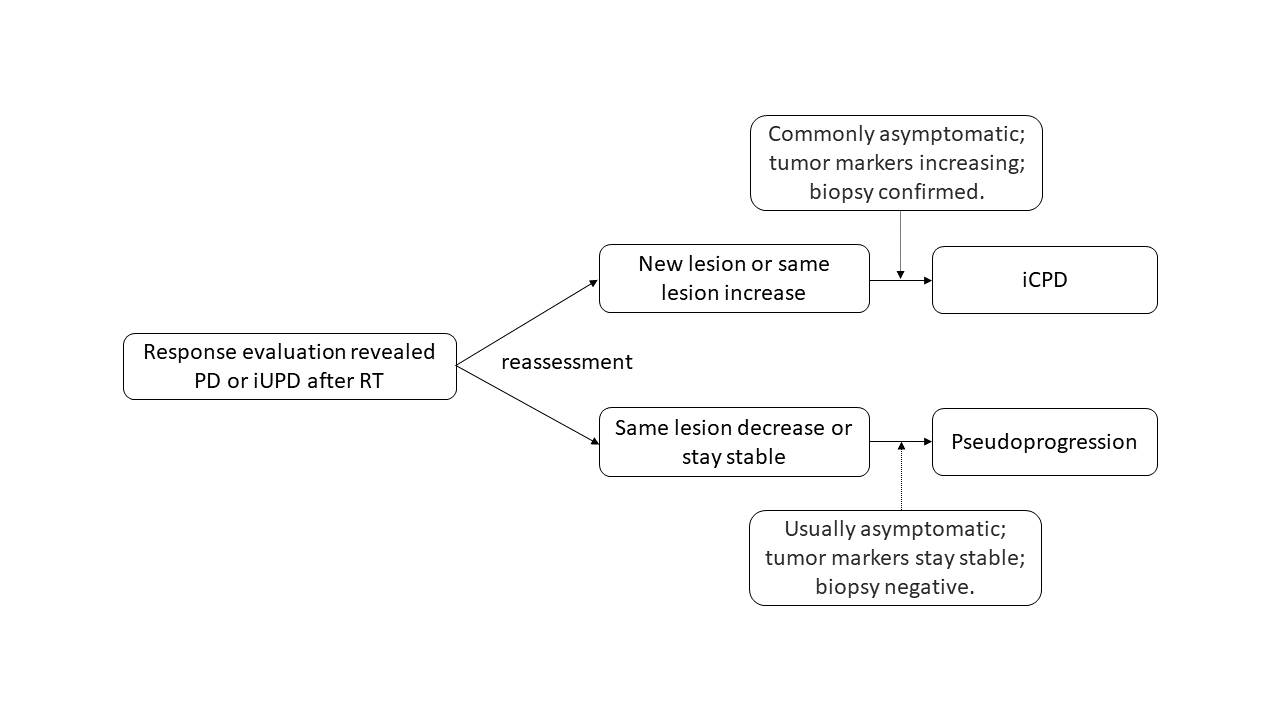

Supplement: Supplementary Figure 1 — A flow chart of differentiating pseudoprogression and real progression. PD, progressive disease; iUPD, immune unconfirmed progressive disease; iCPD, immune confirmed progressive disease. [file Image_1.png]
